# *catena*-Poly[[trimeth­yl(4-sulfanylphen­yl)aza­nium] [(chloridocadmate)-di-μ-chlorido]]

**DOI:** 10.1107/S1600536811050513

**Published:** 2011-11-30

**Authors:** Xiao-Yan Tang, Jian-Ping Lang

**Affiliations:** a College of Chemistry & Materials Engineering, Jiangsu Laboratory of Advanced Functional Materials, Changshu Institute of Technology, Changshu, 215500, Jiangsu, People’s Republic of China; b Key Laboratory of Organic Synthesis of Jiangsu Province, School of Chemistry and Chemical Engineering, Suzhou Uinversity, Suzhou 215123, Jiangsu, People’s Republic of China

## Abstract

The title compound, {(C_9_H_14_NS)[CdCl_3_]}_*n*_, consists of a linear [CdCl_3_]*_n_^n^* ^−^ polyanion and a trimeth­yl(4-sulfanylphen­yl)aza­nium cation. The Cd^II^ atom is penta­coordinated by four μ_2_-Cl atoms and one terminal Cl atom in a trigonal–bipyramidal geometry. The trigonal–bipyramidal units are linked by two opposite shared faces, giving rise to infinite [CdCl_3_]_*n*_ chains parallel to the *a* axis. The cations surround the chain and are linked to them by S—H⋯Cl and C—H⋯Cl hydrogen bonds, forming a three-dimensional network.

## Related literature

For the synthesis of trimethyl­ammonium­phenyl-4-thiol hexa­fluorido­phosphate, see: DePamphilis *et al.* (1974 ▶).
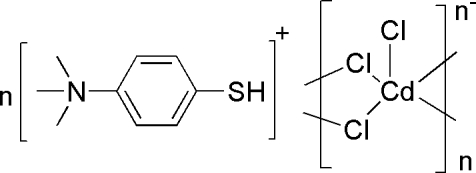


## Experimental

### 

#### Crystal data

(C_9_H_14_NS)[CdCl_3_]
*M* *_r_* = 387.04
Monoclinic, 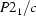

*a* = 7.3207 (15) Å
*b* = 20.971 (4) Å
*c* = 9.1613 (18) Å
β = 103.96 (3)°
*V* = 1364.9 (5) Å^3^
*Z* = 4
Mo *K*α radiation
μ = 2.31 mm^−1^
*T* = 223 K
0.50 × 0.30 × 0.20 mm

#### Data collection

Rigaku Mercury diffractometer
Absorption correction: multi-scan (*CrystalClear*; Rigaku/MSC, 2001 ▶) *T*_min_ = 0.442, *T*_max_ = 0.635
12984 measured reflections
2491 independent reflections
2424 reflections with *I* > 2σ(*I*)
*R*_int_ = 0.027

#### Refinement

*R*[*F* ^2^ > 2σ(*F* ^2^)] = 0.026
*wR*(*F* ^2^) = 0.069
*S* = 1.11
2491 reflections
137 parameters
H-atom parameters constrained
Δρ_max_ = 0.56 e Å^−3^
Δρ_min_ = −0.79 e Å^−3^

### 

Data collection: *CrystalClear* (Rigaku/MSC, 2001 ▶); cell refinement: *CrystalClear*; data reduction: *CrystalStructure* (Rigaku/MSC, 2004 ▶); program(s) used to solve structure: *SHELXS97* (Sheldrick, 2008 ▶); program(s) used to refine structure: *SHELXL97* (Sheldrick, 2008 ▶); molecular graphics: *SHELXTL* (Sheldrick, 2008 ▶); software used to prepare material for publication: *SHELXL97*.

## Supplementary Material

Crystal structure: contains datablock(s) I, global. DOI: 10.1107/S1600536811050513/ng5241sup1.cif

Structure factors: contains datablock(s) I. DOI: 10.1107/S1600536811050513/ng5241Isup2.hkl

Additional supplementary materials:  crystallographic information; 3D view; checkCIF report

## Figures and Tables

**Table 1:** Hydrogen-bond geometry (Å, °)

*D*—H⋯*A*	*D*—H	H⋯*A*	*D*⋯*A*	*D*—H⋯*A*
S1—H1⋯Cl1^i^	1.20	2.55	3.746	180
C8—H8*B*⋯Cl2^ii^	0.97	2.72	3.640 (3)	158

Symmetry codes: (i) 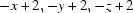; (ii) 
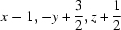
.

**Fig. 1.**
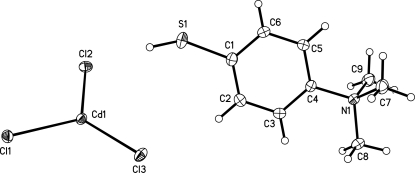
ORTEP plot of complex (I) at the 30% probability level. Hydrogen atoms are drawn as spheres of arbitrary radii.

**Fig. 2.**
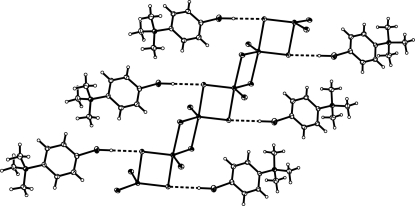
One dimensional structure formed by S—H···Cl interactions.

**Fig. 3.**
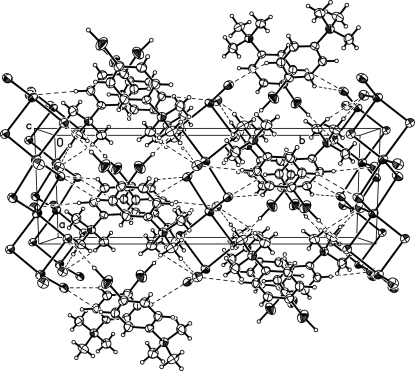
Three dimensional networks formed by S—H···Cl and C—H···Cl interactions.

## supplementary crystallographic information

### Experimental

The synthesis of trimethylammoniumphenyl-4-thiol hexafluorophosphate was according to the literature procedure (DePamphilis *et al.*, 1974). To a suspension containing TabHPF_6_ (0.125 mg, 0.4 mmol) in MeOH (15 ml) was added Et_3_N (0.5 ml). The resulting colorless solution was then treated with a solution of CdCl_2_^.^2.5H_2_O (0.091 g, 0.4 mmol) in MeOH (10 ml). The mixture was stirred at room temperature for 1 h and treated with HCl to adjust the pH to 3, and then filtered. Diethyl ether (20 ml) was allowed to diffuse onto the filtrate. After standing it at ambient temperature for one week, colorless block crystals of (I) were formed. Yield: 0.100 g (65% based on Cd). The crystal used for the crystal structure determination was obtained directly from the above preparation. Analysis, found: C, 27.52; H, 3.31; N, 4.02%. calculated. for C_9_H_14_NSCdCl_3_: C, 27.93; H, 3.65; N, 3.62%.

### Refinement

Carbon-bond H atoms were positioned geometrically (C—H = 0.94 Å for methylene group and C—H = 0.97 Å for methyl group), and were included in the refinement in the riding mode approximation, with *U*_iso_(H) = 1.2*U*_eq_(C)for methylene group and *U*_iso_(H) = 1.5*U*_eq_(C)for methyl group. The H atom attached to atom S1 was located in a difference Fourier map and refined isotropically without constraints with *U*_iso_(H) values fixed at 1.2*U*_eq_(S)].

### Crystal data

**Table:**

(C_9_H_14_NS)[CdCl_3_]	*F*(000) = 760
*M**_r_* = 387.04	*D*_x_ = 1.883 Mg m^−^^3^
Monoclinic, *P*2_1_/*c*	Mo *K*α radiation, λ = 0.71073 Å
Hall symbol: -P 2ybc	Cell parameters from 3007 reflections
*a* = 7.3207 (15) Å	θ = 3.0–25.4°
*b* = 20.971 (4) Å	µ = 2.31 mm^−^^1^
*c* = 9.1613 (18) Å	*T* = 223 K
β = 103.96 (3)°	Block, colorless
*V* = 1364.9 (5) Å^3^	0.50 × 0.30 × 0.20 mm
*Z* = 4	

### Data collection

**Table:**

Rigaku Mercury diffractometer	2491 independent reflections
Radiation source: fine-focus sealed tube	2424 reflections with *I* > 2σ(*I*)
graphite	*R*_int_ = 0.027
ω scans	θ_max_ = 25.4°, θ_min_ = 3.0°
Absorption correction: multi-scan (*CrystalClear*; Rigaku/MSC2001)	*h* = −8→7
*T*_min_ = 0.442, *T*_max_ = 0.635	*k* = −22→25
12984 measured reflections	*l* = −11→11

### Refinement

**Table:**

Refinement on *F*^2^	Secondary atom site location: difference Fourier map
Least-squares matrix: full	Hydrogen site location: inferred from neighbouring sites
*R*[*F*^2^ > 2σ(*F*^2^)] = 0.026	H-atom parameters constrained
*wR*(*F*^2^) = 0.069	*w* = 1/[σ^2^(*F*_o_^2^) + (0.038*P*)^2^ + 0.9902*P*] where *P* = (*F*_o_^2^ + 2*F*_c_^2^)/3
*S* = 1.11	(Δ/σ)_max_ < 0.001
2491 reflections	Δρ_max_ = 0.56 e Å^−^^3^
137 parameters	Δρ_min_ = −0.79 e Å^−^^3^
0 restraints	Extinction correction: *SHELXL97* (Sheldrick, 2008), Fc^*^=kFc[1+0.001xFc^2^λ^3^/sin(2θ)]^-1/4^
Primary atom site location: structure-invariant direct methods	Extinction coefficient: 0.0059 (5)

### Special details

**Table:**

Geometry. All e.s.d.'s (except the e.s.d. in the dihedral angle between two l.s. planes) are estimated using the full covariance matrix. The cell e.s.d.'s are taken into account individually in the estimation of e.s.d.'s in distances, angles and torsion angles; correlations between e.s.d.'s in cell parameters are only used when they are defined by crystal symmetry. An approximate (isotropic) treatment of cell e.s.d.'s is used for estimating e.s.d.'s involving l.s. planes.
Refinement. Refinement of *F*^2^ against ALL reflections. The weighted *R*-factor *wR* and goodness of fit *S* are based on *F*^2^, conventional *R*-factors *R* are based on *F*, with *F* set to zero for negative *F*^2^. The threshold expression of *F*^2^ > σ(*F*^2^) is used only for calculating *R*-factors(gt) *etc*. and is not relevant to the choice of reflections for refinement. *R*-factors based on *F*^2^ are statistically about twice as large as those based on *F*, and *R*– factors based on ALL data will be even larger.

### Fractional atomic coordinates and isotropic or equivalent isotropic displacement parameters (Å^2^)

**Table:**

	*x*	*y*	*z*	*U*_iso_*/*U*_eq_	
Cd1	0.73386 (3)	0.993777 (10)	0.92593 (2)	0.02485 (11)	
Cl1	0.95247 (10)	1.08537 (3)	0.99446 (8)	0.02873 (17)	
Cl2	0.68785 (10)	0.96938 (3)	0.66114 (7)	0.03076 (18)	
Cl3	0.59419 (8)	0.93630 (3)	1.11069 (7)	0.03094 (18)	
S1	0.67638 (8)	0.79389 (3)	0.84461 (7)	0.0484 (2)	
H1	0.7952	0.8326	0.8962	0.058*	
C1	0.5039 (4)	0.74912 (14)	0.9045 (3)	0.0285 (6)	
C2	0.3807 (4)	0.77654 (14)	0.9805 (3)	0.0319 (6)	
H2	0.3876	0.8205	1.0016	0.038*	
C3	0.2478 (4)	0.73917 (14)	1.0253 (3)	0.0303 (6)	
H3	0.1638	0.7577	1.0760	0.036*	
C4	0.2392 (4)	0.67468 (13)	0.9952 (3)	0.0238 (6)	
C5	0.3603 (4)	0.64695 (14)	0.9182 (3)	0.0278 (6)	
H5	0.3540	0.6030	0.8977	0.033*	
C6	0.4898 (4)	0.68458 (14)	0.8721 (3)	0.0301 (6)	
H6	0.5700	0.6662	0.8177	0.036*	
C7	0.2022 (4)	0.58027 (15)	1.1450 (4)	0.0350 (7)	
H7A	0.2689	0.5534	1.0889	0.053*	
H7B	0.1119	0.5548	1.1813	0.053*	
H7C	0.2914	0.5991	1.2297	0.053*	
C8	−0.0195 (4)	0.66781 (15)	1.1295 (3)	0.0342 (7)	
H8A	0.0607	0.6878	1.2174	0.051*	
H8B	−0.1042	0.6381	1.1610	0.051*	
H8C	−0.0922	0.7003	1.0654	0.051*	
C9	−0.0285 (4)	0.60310 (16)	0.9076 (3)	0.0357 (7)	
H9A	−0.0869	0.6365	0.8389	0.054*	
H9B	−0.1251	0.5785	0.9381	0.054*	
H9C	0.0436	0.5754	0.8578	0.054*	
N1	0.1003 (3)	0.63245 (11)	1.0439 (2)	0.0250 (5)	

### Atomic displacement parameters (Å^2^)

**Table:**

	*U*^11^	*U*^22^	*U*^33^	*U*^12^	*U*^13^	*U*^23^
Cd1	0.02433 (16)	0.02926 (16)	0.02258 (16)	−0.00438 (7)	0.00880 (10)	−0.00082 (7)
Cl1	0.0240 (3)	0.0261 (4)	0.0358 (4)	−0.0021 (3)	0.0065 (3)	−0.0020 (3)
Cl2	0.0328 (4)	0.0364 (4)	0.0230 (3)	0.0030 (3)	0.0066 (3)	−0.0025 (3)
Cl3	0.0280 (4)	0.0330 (4)	0.0359 (4)	0.0061 (3)	0.0157 (3)	0.0131 (3)
S1	0.0499 (5)	0.0405 (5)	0.0632 (6)	−0.0145 (4)	0.0301 (4)	0.0025 (4)
C1	0.0287 (15)	0.0301 (15)	0.0267 (14)	−0.0036 (11)	0.0066 (11)	0.0032 (11)
C2	0.0352 (16)	0.0247 (15)	0.0366 (16)	−0.0010 (12)	0.0105 (12)	−0.0012 (12)
C3	0.0336 (16)	0.0282 (16)	0.0322 (15)	0.0008 (12)	0.0141 (12)	−0.0046 (12)
C4	0.0220 (13)	0.0257 (14)	0.0240 (14)	−0.0025 (11)	0.0065 (10)	0.0012 (11)
C5	0.0310 (15)	0.0246 (15)	0.0297 (15)	−0.0001 (11)	0.0112 (12)	−0.0034 (11)
C6	0.0296 (15)	0.0310 (16)	0.0337 (16)	0.0005 (12)	0.0152 (12)	−0.0025 (12)
C7	0.0374 (17)	0.0319 (17)	0.0379 (17)	0.0022 (13)	0.0133 (13)	0.0094 (13)
C8	0.0333 (16)	0.0372 (17)	0.0377 (17)	−0.0009 (13)	0.0197 (13)	−0.0029 (13)
C9	0.0304 (16)	0.0440 (18)	0.0339 (16)	−0.0111 (13)	0.0101 (12)	−0.0082 (13)
N1	0.0265 (12)	0.0262 (12)	0.0245 (11)	−0.0007 (9)	0.0102 (9)	0.0000 (9)

### Geometric parameters (Å, °)

**Table:**

Cd1—Cl2	2.4219 (8)	C4—N1	1.496 (3)
Cd1—Cl1	2.4819 (8)	C5—C6	1.375 (4)
Cd1—Cl3	2.4896 (7)	C5—H5	0.9400
Cd1—Cl3^i^	2.7643 (7)	C6—H6	0.9400
Cd1—Cl1^ii^	2.7842 (9)	C7—N1	1.509 (4)
Cl1—Cd1^ii^	2.7842 (9)	C7—H7A	0.9700
Cl3—Cd1^i^	2.7643 (7)	C7—H7B	0.9700
S1—C1	1.764 (3)	C7—H7C	0.9700
S1—H1	1.1999	C8—N1	1.505 (4)
C1—C6	1.384 (4)	C8—H8A	0.9700
C1—C2	1.390 (4)	C8—H8B	0.9700
C2—C3	1.386 (4)	C8—H8C	0.9700
C2—H2	0.9400	C9—N1	1.503 (4)
C3—C4	1.379 (4)	C9—H9A	0.9700
C3—H3	0.9400	C9—H9B	0.9700
C4—C5	1.387 (4)	C9—H9C	0.9700
			
Cl2—Cd1—Cl1	110.02 (3)	C5—C6—C1	121.1 (3)
Cl2—Cd1—Cl3	126.73 (3)	C5—C6—H6	119.4
Cl1—Cd1—Cl3	123.24 (3)	C1—C6—H6	119.4
Cl2—Cd1—Cl3^i^	94.80 (3)	N1—C7—H7A	109.5
Cl1—Cd1—Cl3^i^	96.23 (3)	N1—C7—H7B	109.5
Cl3—Cd1—Cl3^i^	81.49 (3)	H7A—C7—H7B	109.5
Cl2—Cd1—Cl1^ii^	92.47 (3)	N1—C7—H7C	109.5
Cl1—Cd1—Cl1^ii^	87.38 (3)	H7A—C7—H7C	109.5
Cl3—Cd1—Cl1^ii^	88.93 (3)	H7B—C7—H7C	109.5
Cl3^i^—Cd1—Cl1^ii^	170.23 (2)	N1—C8—H8A	109.5
Cd1—Cl1—Cd1^ii^	92.62 (3)	N1—C8—H8B	109.5
Cd1—Cl3—Cd1^i^	98.51 (3)	H8A—C8—H8B	109.5
C1—S1—H1	138.0	N1—C8—H8C	109.5
C6—C1—C2	119.2 (3)	H8A—C8—H8C	109.5
C6—C1—S1	118.4 (2)	H8B—C8—H8C	109.5
C2—C1—S1	122.4 (2)	N1—C9—H9A	109.5
C3—C2—C1	120.1 (3)	N1—C9—H9B	109.5
C3—C2—H2	120.0	H9A—C9—H9B	109.5
C1—C2—H2	120.0	N1—C9—H9C	109.5
C4—C3—C2	119.8 (3)	H9A—C9—H9C	109.5
C4—C3—H3	120.1	H9B—C9—H9C	109.5
C2—C3—H3	120.1	C4—N1—C9	109.2 (2)
C3—C4—C5	120.6 (3)	C4—N1—C8	112.8 (2)
C3—C4—N1	121.5 (2)	C9—N1—C8	107.9 (2)
C5—C4—N1	117.9 (2)	C4—N1—C7	109.9 (2)
C6—C5—C4	119.2 (3)	C9—N1—C7	109.3 (2)
C6—C5—H5	120.4	C8—N1—C7	107.6 (2)
C4—C5—H5	120.4		

Symmetry codes: (i) −*x*+1, −*y*+2, −*z*+2; (ii) −*x*+2, −*y*+2, −*z*+2.

### Hydrogen-bond geometry (Å, °)

**Table:**

*D*—H···*A*	*D*—H	H···*A*	*D*···*A*	*D*—H···*A*
S1—H1···Cl1^ii^	1.20	2.55	3.746	180.
C8—H8B···Cl2^iii^	0.97	2.72	3.640 (3)	158.

Symmetry codes: (ii) −*x*+2, −*y*+2, −*z*+2; (iii) *x*−1, −*y*+3/2, *z*+1/2.
